# Passive immunotherapy against Aβ in aged APP-transgenic mice reverses cognitive deficits and depletes parenchymal amyloid deposits in spite of increased vascular amyloid and microhemorrhage

**DOI:** 10.1186/1742-2094-1-24

**Published:** 2004-12-08

**Authors:** Donna M Wilcock, Amyn Rojiani, Arnon Rosenthal, Sangeetha Subbarao, Melissa J Freeman, Marcia N Gordon, Dave Morgan

**Affiliations:** 1 Alzheimer's Research Laboratory, University of South Florida, Department of Pharmacology, 12901 Bruce B Downs Blvd, Tampa, Florida 33612, USA; 2 Alzheimer's Research Laboratory, University of South Florida, Department of Interdisciplinary Oncology, 12901 Bruce B Downs Blvd, Tampa, Florida 33612, USA; 3 Rinat Neuroscience Corp., 3155 Porter Drive, Palo Alto, California 94304, USA

## Abstract

**Background:**

Anti-Aβ immunotherapy in transgenic mice reduces both diffuse and compact amyloid deposits, improves memory function and clears early-stage phospho-tau aggregates. As most Alzheimer disease cases occur well past midlife, the current study examined adoptive transfer of anti-Aβ antibodies to 19- and 23-month old APP-transgenic mice.

**Methods:**

We investigated the effects of weekly anti-Aβ antibody treatment on radial-arm water-maze performance, parenchymal and vascular amyloid loads, and the presence of microhemorrhage in the brain. 19-month-old mice were treated for 1, 2 or 3 months while 23-month-old mice were treated for 5 months. Only the 23-month-old mice were subject to radial-arm water-maze testing.

**Results:**

After 3 months of weekly injections, this passive immunization protocol completely reversed learning and memory deficits in these mice, a benefit that was undiminished after 5 months of treatment. Dramatic reductions of diffuse Aβ immunostaining and parenchymal Congophilic amyloid deposits were observed after five months, indicating that even well-established amyloid deposits are susceptible to immunotherapy. However, cerebral amyloid angiopathy increased substantially with immunotherapy, and some deposits were associated with microhemorrhage. Reanalysis of results collected from an earlier time-course study demonstrated that these increases in vascular deposits were dependent on the duration of immunotherapy.

**Conclusions:**

The cognitive benefits of passive immunotherapy persist in spite of the presence of vascular amyloid and small hemorrhages. These data suggest that clinical trials evaluating such treatments will require precautions to minimize potential adverse events associated with microhemorrhage.

## Background

Alzheimer's disease is characterized not only by the presence of parenchymal amyloid deposits and intracellular tangles but also by the presence of amyloid deposits in the vasculature, a condition referred to as cerebral amyloid angiopathy (CAA). The CAA observed in both Alzheimer's disease patients [1] and some of the transgenic mouse models [2] is primarily composed of the shorter form of amyloid beta (Aβ), Aβ_1–40_, while the majority of amyloid deposits in the parenchyma are composed of Aβ_1–42_, although the compact amyloid deposits also contain Aβ_1–40_.

Anti-Aβ immunotherapy has been considered as a potential treatment for Alzheimer's disease for some time [3,4]. Active immunization with a vaccine including Aβ_1–42 _fibrils progressed to human clinical trials where its administration was suspended due to meningoencephalitits in a subset of patients [5]. To date there have been pathology reports on two patients who participated in the trial and subsequently died [6,7]. Both reports note that while the numbers of parenchymal amyloid deposits appeared lower than expected in these cases, the CAA in these patients did not appear outside the normal range for Alzheimer's disease. In addition, one report mentioned multiple cortical hemorrhages and the presence of hemosiderin around the CAA vessels [7].

Given the adverse reactions to the active immunization, the irreversibility of such procedures and the variable antibody response to vaccines in older individuals [8], passive immunization against the Aβ peptide emerged as an alternative immunotherapeutic strategy. Studies in young and middle aged APP-transgenic mice have reported significant amyloid reductions with passive immunization [9-11]. Such treatments also demonstrate rapid improvements of memory function in APP-transgenic mice, sometimes without detectable reductions in amyloid [12-14]. Most recently, intracranial administration of anti-Aβ antibodies has been shown to not only remove Aβ but also clear, early-stage, hyperphosphorylated-tau aggregates [15]. Importantly, in the only prior study evaluating adoptive antibody transfer in older APP-transgenic mice, Pfeifer *et al*. [16] reported a doubling of cerebral microhemorrhages associated with significant reductions in amyloid burden after administration of an N-terminal specific anti-Aβ antibody.

## Materials and Methods

### Experiment design

Mice derived from APP Tg2576 mice were obtained from our breeding program at University of South Florida started in 1996 [17]. For the 5-month treatment study, 13 APP-transgenic mice, aged 23 months, were assigned to one of two groups. The first group received weekly intraperitoneal anti-Aβ antibody injections (antibody 2286; mouse-monoclonal anti-human Aβ_28–40 _IgG1; Rinat Neurosciences, Palo Alto, CA) for a period of 5 months (*n *= 6). The second group received weekly intraperitoneal anti-AMN antibody (2906; mouse-monoclonal anti-*Drosophila *amnesiac protein IgG1; Rinat Neurosciences, Palo Alto, CA) injections for a period of 5 months (*n *= 7). Seven nontransgenic mice were also assigned to one of two groups. The first group received weekly intraperitoneal anti-Aβ antibody injections for a period of 5 months (*n *= 4). The second group received weekly intraperitoneal anti-AMN antibody injections for a period of 5 months (*n *= 3).

For the time course study of 1-, 2- or 3-month treatment, 22 APP-transgenic mice aged 19 months were assigned to one of four experimental groups, as described previously [14]. The first three groups received weekly intraperitoneal anti-Aβ antibody injections for 3 months, 2 months or 1 month, ending when all mice were 22 months of age. The fourth group received weekly intraperitoneal anti-AMN antibody injections for 3 months.

### Behavioral analysis

Following 3 and 5 months of treatment, the mice from the 5-month study were subjected to a two-day radial-arm water-maze paradigm. The apparatus was a 6-arm maze as described previously [18]. On day one, 15 trials were run in three blocks of 5. A cohort of 4 mice were run sequentially for each block (i.e., each of 4 mice get trial one, then the same mice get trial two, etc.). After each 5-trial block, a second cohort of mice was run permitting an extended rest period before mice were exposed to the second block of 5 trials. The goal arm was different for each mouse in a cohort to minimize odor cues. The start arm was varied for each trial, with the goal arm remaining constant for a given individual for both days. For the first 11 trials, the platform was alternately visible then hidden (hidden for the last 4 trials). On day two, the mice were run in exactly the same manner as day one except that the platform was hidden forall trials. The number of errors (incorrect arm entries) was measured in a one-minute time frame. As standard practice, mice failing to make an arm choice in 20 seconds are assigned one error, but no mice in this study had to be assigned an error in this manner. The same individual administered the antibody treatments and placed mice in the radial-arm water maze. Due to the numbers of mice in the study the researcher was unaware of treatment group identity of each mouse. Also, the dependent measures in the radial-arm water-maze task are quantitative, not evaluative, so the potential for tester bias is reduced. In order to minimize the influence of individual trial variability, each mouse's errors for 3 consecutive trials were averaged producing 5 data points for each day, which were analyzed statistically by ANOVA using StatView (SAS Institute Inc., NC).

### Tissue preparation and histology

On the day of sacrifice mice were weighed, overdosed with 100 mg/kg Nembutal (Abbott laboratories, North Chicago, IL), and then intracardially perfused with 25 mL of 0.9% sodium chloride. Brains were rapidly removed, and the left half of the brain was immersion fixed for 24 h in freshly prepared 4% paraformaldehyde in 100 mM KPO_4 _(pH 7.2) for histopathology. The hemi-brains were then incubated for 24 h in 10%, 20% and 30% sucrose sequentially for cyroprotection. Horizontal sections of 25 μ thickness were collected using a sliding microtome and stored at 4°C in Dulbecco's phosphate-buffered saline with sodium azide (pH 7.2) to prevent microbial growth. A series of 8 equally spaced tissue sections 600 μ apart were randomly selected spanning the entire brain and stained using free-floating immunohistochemistry for total Aβ (rabbit polyclonal anti-pan Aβ; Biosource, Camarillo, CA, 1:10,000) as previously described [2,14]. A second series of tissue sections 600 μm apart were stained using 0.2% Congo red in NaCl-saturated 80% ethanol. Another set of sections were also mounted and stained for hemosiderin using 2% potassium ferrocyanide in 2% hydrochloric acid for 15 min, followed by a counterstain in a 1% neutral red solution for 10 min. Quantification of Congo red staining and Aβ immunohistochemistry was performed using the Image-Pro Plus (Media Cybernetics, Silver Spring, MD) to analyze the percent area occupied by positive stain. One region of the frontal cortex and three regions of the hippocampus were analyzed (to ensure that there was no regional bias in the hippocampal values). The initial analysis of Congo red was performed to give a total value. A second analysis was performed after manually editing out all of the parenchymal amyloid deposits to yield a percent area restricted to vascular Congo red staining. To estimate the parenchymal area of Congo red, we subtracted the vascular amyloid values from the total percentage. For the hemosiderin stain the numbers of Prussian blue-positive sites were counted on all sections and the average number of sites per section calculated. Looking at the sections at a low magnification we were able to observe a qualitative differences between animals; however, the percent area was so low that many fields contained no positive stain. Eight equally spaced sections were examined and the number of positive profiles was determined and averaged to a per-section value. To assess possible treatment-related differences, the values for each treatment group were analyzed by one-way ANOVA followed by Fisher's LSD means comparisons.

## Results

### Reversal of cognitive deficits by passive amyloid immunotherapy

**Figure 1:**
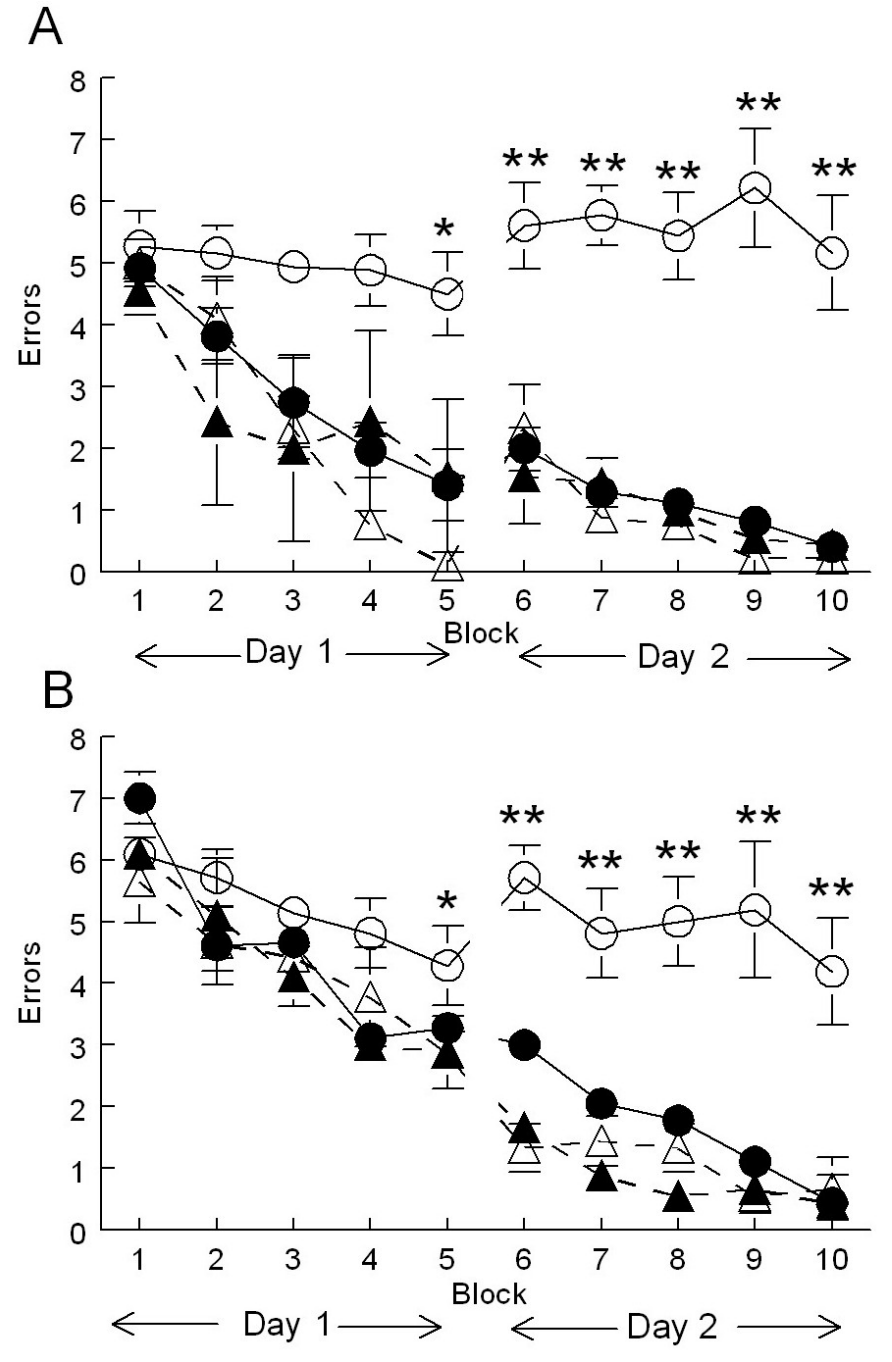
Spatial learning deficits in APP-transgenic mice were reversed following 3 and 5 months of immunization. Mice were tested in a two-day version of the radial-arm water maze. Solid lines represent APP-transgenic mice while dashed lines represent nontransgenic mice. Open symbols indicate anti-AMN, control-antibody treatment (○: APP-transgenic, control antibody; △: nontransgenic, control antibody) while closed symbols indicate anti-Aβ antibody treatment (●: APP-transgenic, Aβ antibody; ▲: nontransgenic, Aβ antibody). Panel A shows mean number of errors made over the two-day trial period following 3 months of immunization. Each data point is the average of 3 trials. Panel B shows the mean number of errors made over the 2-day trial period following 5 months of immunization. For both graphs * indicates *p *< 0.05, ** indicates *p *< 0.001 when the APP-transgenic mice receiving control antibody are compared with the remaining groups.

The radial-arm water-maze task detects spatial learning and memory deficits in transgenic mouse models [18,19]. We treated 23-month-old mice for 5 months with anti-Aβ antibody 2286 or control antibody 2906 (against a *Drosophila*-specific protein) and tested them for spatial navigation learning in a two-day version of the radial-arm water maze after 3 months of treatment and, using a new platform location, again after 5 months of treatment. At both testing times we found that APP-transgenic mice treated with the control antibody failed to learn platform location over two days of testing and were significantly impaired compared to the nontransgenic mice treated with either antibody (Fig. 1). However, APP-transgenic mice administered the anti-Aβ antibodies demonstrated a complete reversal of the impairment observed in the control-treated APP-transgenic mice, ending day two with a mean performance near 0.5 errors per trial (Fig. 1). Although learning at the later time point, when the mice were 28 months of age, may have been slightly slower for all groups, there was no impairment of the anti-Aβ antibody-treated APP.

### Passive amyloid immunotherapy clears parenchymal Aβ deposits, but increases vascular amyloid

**Figure 2:**
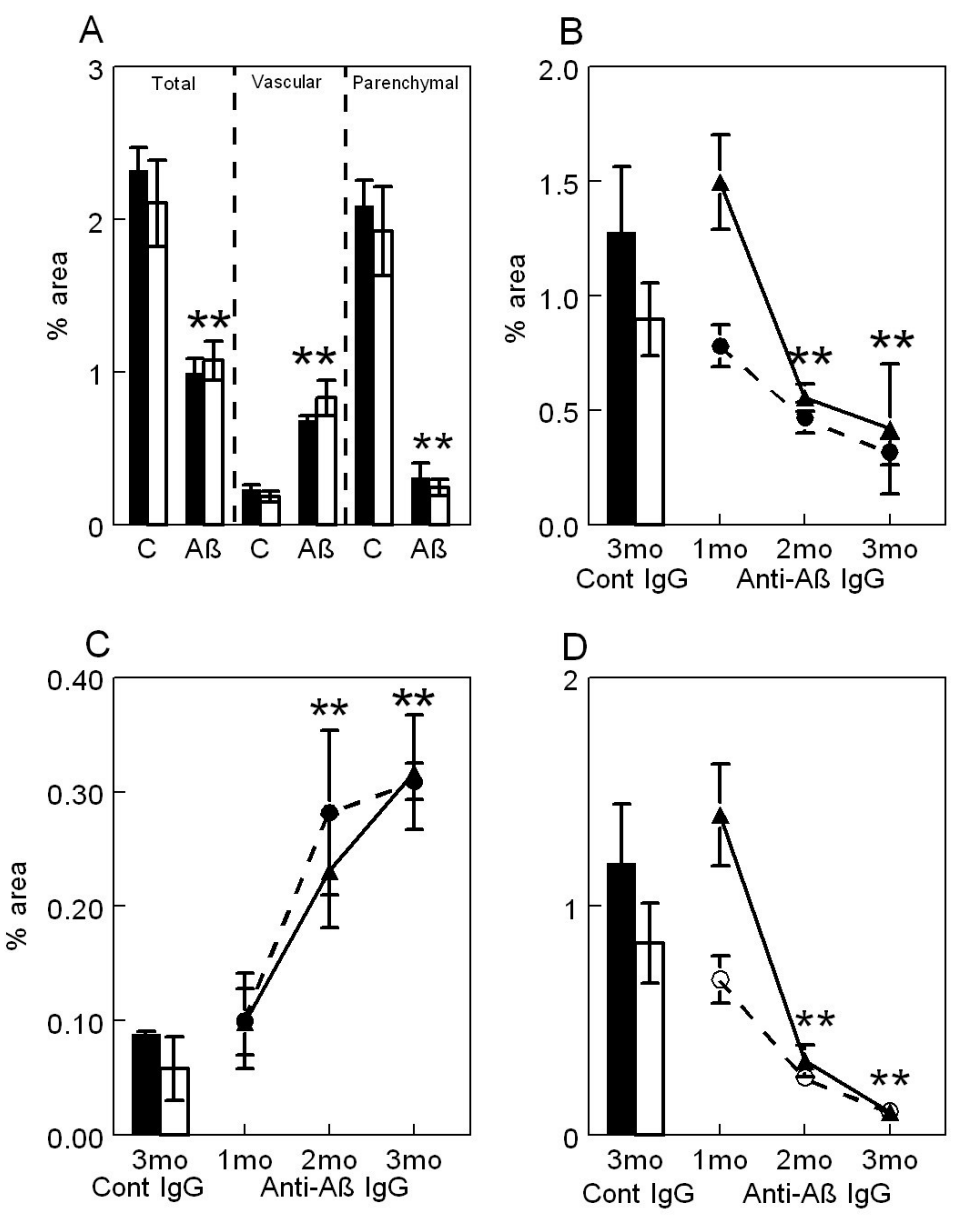
Passive immunization with anti-Aβ antibodies decreases total and parenchymal amyloid loads while increasing vascular amyloid in frontal cortex and hippocampus of APP-transgenic mice. Panel A shows total amyloid load measured with Congo red, vascular amyloid load and parenchymal amyloid load from APP-transgenic mice administered control IgG (C) or anti-Aβ IgG (Aβ) for a period of 5 months. Panels B-D show total amyloid load (Panel B), vascular amyloid load (Panel C) and parenchymal amyloid load (Panel D) from APP-transgenic mice administered control IgG for 3 months (Cont IgG) or anti-Aβ IgG for a period of 1, 2, or 3 months (Anti-Aβ IgG). For all panels, the solid bar and solid line represent values from the frontal cortex, while the open bar and dashed line represent values from the hippocampus. ** *p *< 0.01.

In a prior experiment examining the effects of passive anti-Aβ immunotherapy for 1, 2 or 3 months in APP-transgenic mice killed at 21 months of age [14], we found a time-dependent reduction of both Aβ immunostaining of diffuse and fibrillar deposits and Congo-red staining of fibrillar amyloid deposits. In the current study we found a similar reduction in both Aβ immunostaining (Table 1) and total Congo-red staining (Fig. 2A, left panel; *p *< 0.001 frontal cortex and *p *< 0.01 hippocampus) after 5 months of immunotherapy. We noted that the bulk of what remained was vascular amyloid. We then separately analyzed vascular and parenchymal deposits which revealed a near 90% reduction in parenchymal deposits (*p *< 0.001) but a 3–4 fold elevation of vascular Congo-red staining (*p *< 0.0001; Fig. 2A, center and right panels, respectively). We also separately analyzed vascular and parenchymal Congo-red staining on mice from our earlier study [14], treated passively for 1, 2 or 3 months with anti-Aβ or control antibody, and found a similar result. There was a graded reduction in overall Congo-red staining nearing 75% as duration of antibody exposure increased (as reported previously; Fig. 2B). However, when separated into vascular Congo-red deposits and parenchymal deposits, there was an antibody-exposure-time-dependent increase in vascular deposition in both hippocampus and frontal cortex (Fig. 2C; *p *< 0.05 frontal cortex and hippocampus) and a corresponding nearly 90% decrease in parenchymal deposits (Fig. 2D; *p *< 0.001 in frontal cortex and hippocampus).

**Table 1:** Total Aβ load is significantly reduced following 5 months of anti-Aβ antibody treatment. Percent area occupied by positive immunohistochemical stain for Aβ is shown ± standard error of the mean for both the frontal cortex and hippocampus. Also shown is the percent reduction of Aβ observed following anti-Aβ antibody treatment

Region	% area positive for Aβ: control treated	% area positive for Aβ: anti-Aβ treated	% reduction following anti-Aβ antibody treatment
Frontal Cortex	34.855 ± 2.265	9.681 ± 0.754	72
Hippocampus	23.994 ± 0.985	8.212 ± 0.596	66

**Figure 3:**
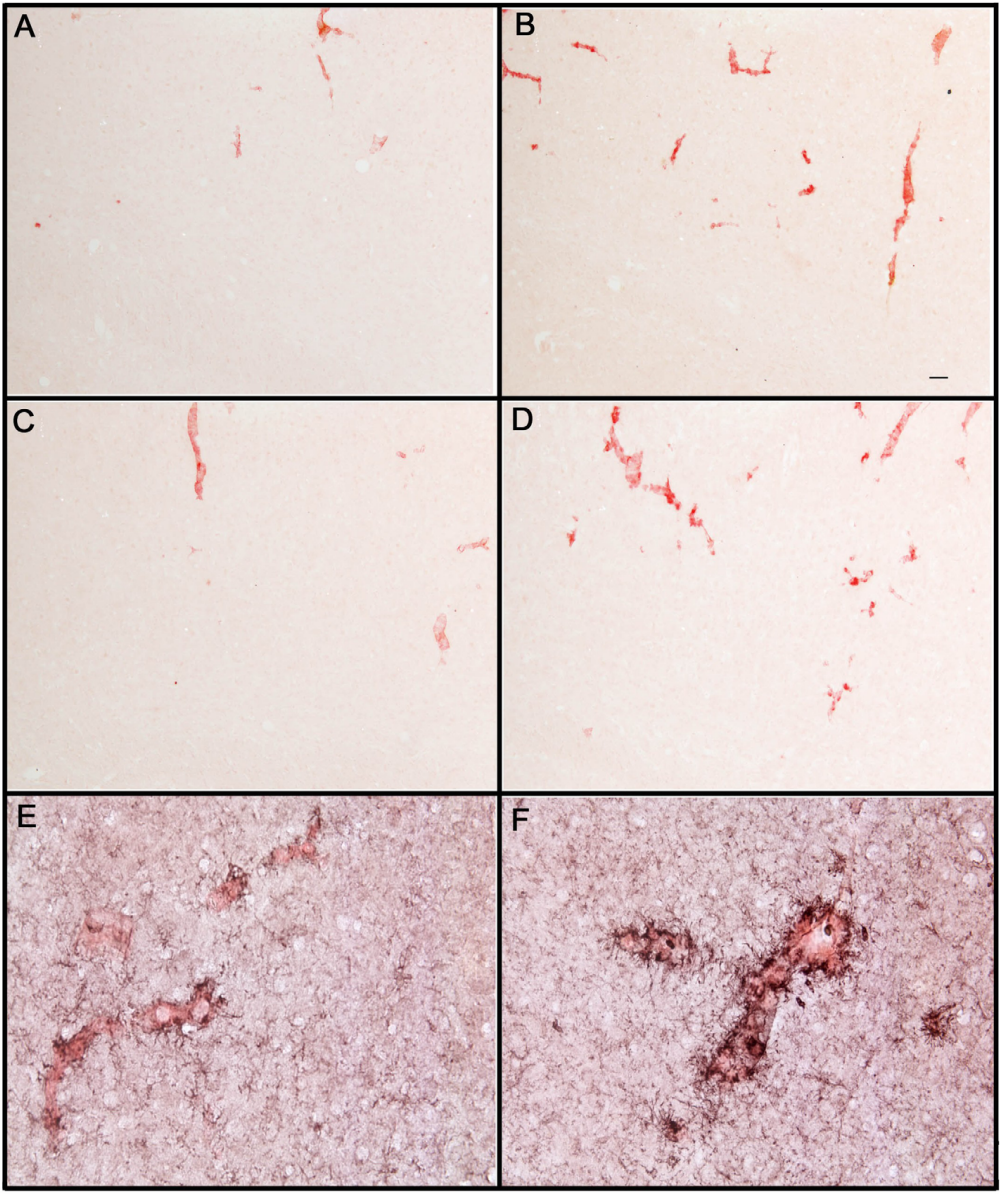
Increased Congo red staining of blood vessels following anti-Aβ antibody administration is associated with activated microglia. Panels A and B are from the frontal cortex of 22-month-old APP-transgenic mice immunized for 3 months with either control antibody (3A) or anti-Aβ antibody (3B). Panels C and D are from the frontal cortex of 28-month-old APP-transgenic mice immunized for 5 months with either control antibody (3C) or anti-Aβ antibody (3D). Panels E and F show a high-magnification image of CD45 immunohistochemistry (black) counterstained with Congo red (red) from 28-month-old APP-transgenic mice immunized for 5 months with either control antibody (Panel E) or anti-Aβ antibody (Panel F). Panels A-D, magnification = 100X. Scale bar in Panel B = 50 μ for panels A-D. Panels E-F, magnification = 200X. Scale bar in Panel E = 25 μm for panels E-F.

These differences were readily observed examining micrographs of sections from these mice. Mice treated with control antibodies revealed occasional cortical vascular amyloid deposits (22 months, Fig. 3A, 28 months, Fig. 3C), while mice administered anti-Aβ antibodies had increased amounts of vascular amyloid staining (3-month treatment, Fig 3B; 5-month treatment, Fig 3D). Those vessels containing amyloid following treatment with anti-Aβ antibody also exhibited apparent increases in microglial activation as measured by CD45 expression (Fig. 3F) compared to mice treated with control antibody (Fig. 3E). Unfortunately, the shifting numbers and sizes of vascular and parenchymal deposits caused by the antibody therapy greatly complicated measurement of microglial activation per vascular deposit area so that this apparent increase in staining intensity could not be quantified accurately.

### Passive amyloid immunotherapy causes increased microhemorrhage

**Figure 4:**
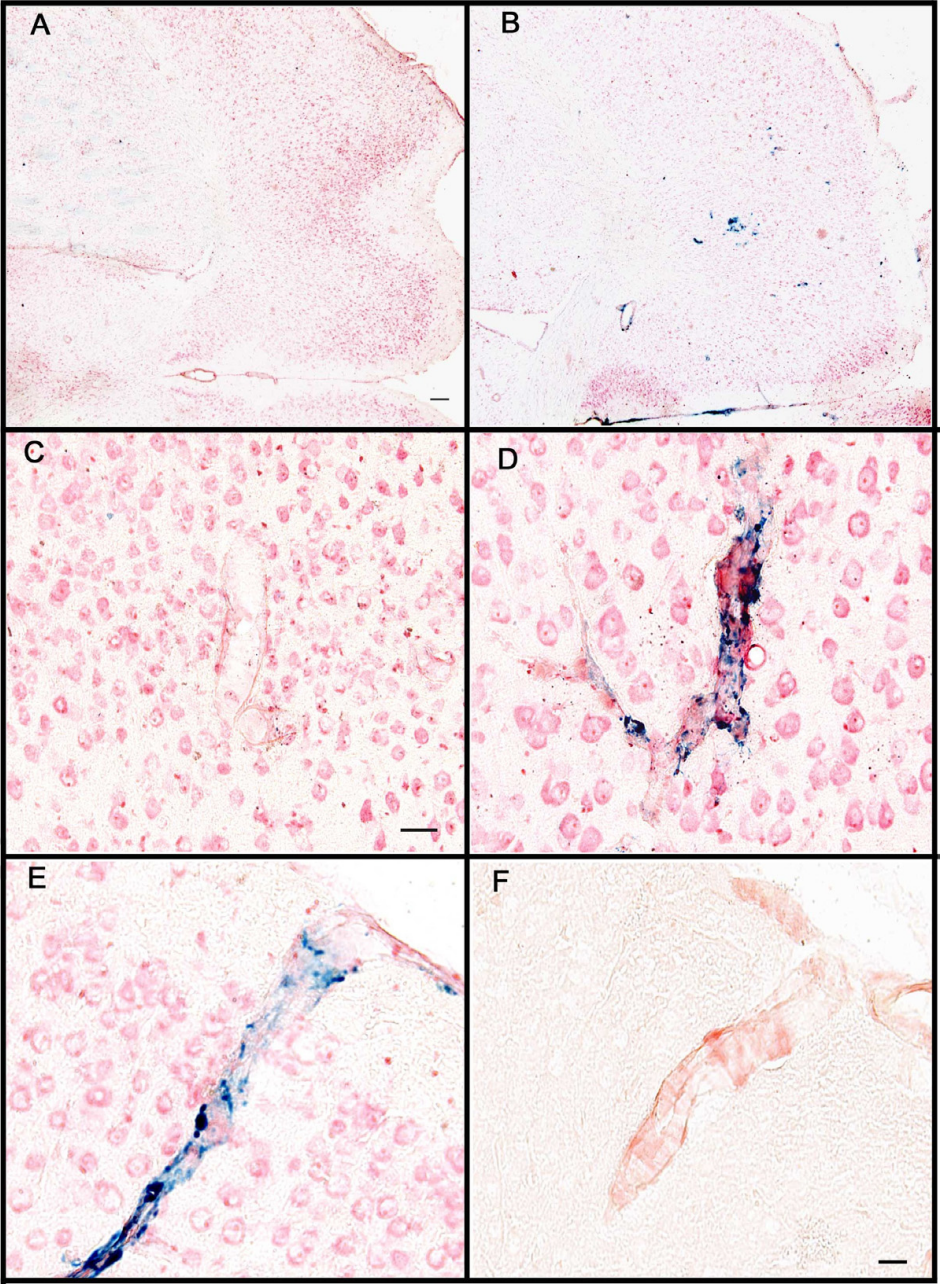
Microhemorrhage associated with CAA following systemic administration of anti-Aβ antibodies. Panels A and B are low magnification images of the frontal cortex of APP-transgenic mice receiving either control antibodies (Panel A) or anti-Aβ antibodies (Panel B) for a period of 5 months. Panels C and D show representative images of amyloid containing vessels stained for Prussian blue (blue), counterstained with neutral red (red), from APP-transgenic mice receiving either control antibodies (Panel C) or anti-Aβ antibodies (Panel D) for a period of 3 months. Panel E shows a blood vessel in the frontal cortex stained for Prussian blue (blue), counterstained with neutral red, from an APP transgenic mouse administered anti-Aβ antibodies for 5 months. Panel F shows the same blood vessel on an adjacent section stained for Congo red, indicating that the blood vessel does in fact contain amyloid. Scale bar panel A = 120 μm for panels A-B. Scale bar panel C = 25 μm for panels C-D. Scale bar in panel F = 25 μm for panels E-F.

We used the Prussian blue histological stain to label hemosiderin, a ferric oxide material produced in the breakdown of hemoglobin. Extravenous blood in the brain leads to microglial phagocytosis of the erythrocytes and breakdown of the hemoglobin within them. These ferric oxide-containing microglia are thus markers of past hemorrhage. In untreated, aged APP-transgenic mice we observed very few profiles positive for Prussian-blue staining in the frontal cortex (section counterstained with neutral red; Fig. 4A). However, following anti-Aβ antibody treatment for 5 months we observed an increase in the number of Prussian-blue profiles in the frontal cortex, which were readily detectable at a low magnification in the microscope (Fig. 4B). In the absence of anti-Aβ treatment, or even when treated with antibody for one month, most vessels did not stain with Prussian blue, and could be identified only using the red counterstain (Fig. 4C). However, even with 3 months of anti-Aβ antibody treatment we observed frequent vessels with associated Prussian-blue staining (Fig 4D). Using adjacent sections stained for Congo red, we confirmed that all vessels showing microhemorrhage contained amyloid (Figs. 4E and 4F; we were unable to double-label Prussian blue-stained sections with either Congo red or thioflavine-S). However, only a minority of vessels containing amyloid demonstrated hemorrhage.

**Figure 5:**
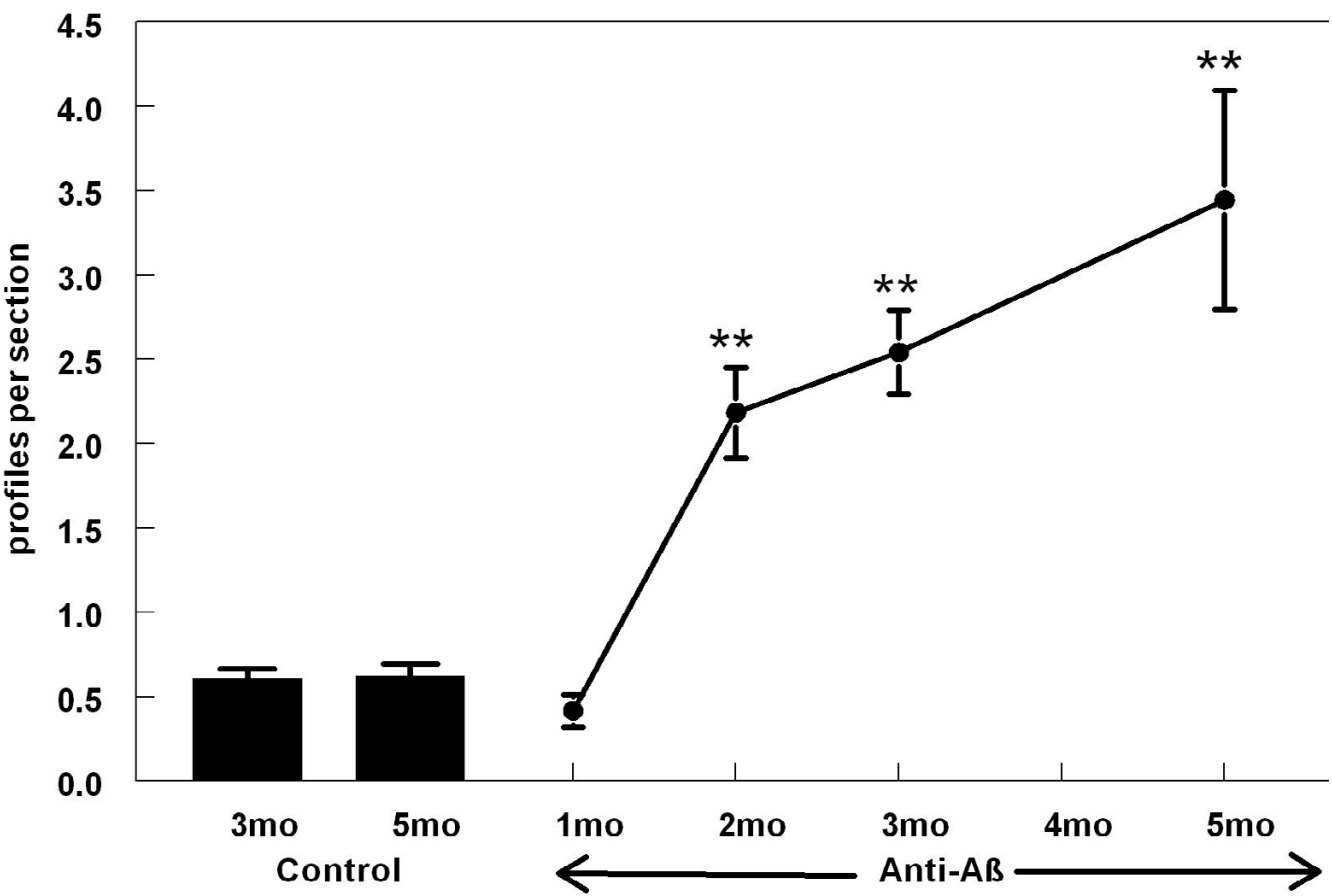
Number of Prussian blue-positive profiles increases with duration of anti-Aβ antibody exposure. The graph shows quantification of the average number of Prussian blue-positive profiles per section from mice administered control IgG for 3 or 5 months (Cont) or anti-Aβ IgG for 1, 2, 3 or 5 months (anti-Aβ). ** *p *< 0.01.

When we counted the number of Prussian blue-positive profiles in those animals receiving control antibody there was an average of one profile per every two sections (Fig. 5) and this number remained the same in both control groups (aged 22 or 28 months). Following treatment with anti-Aβ antibody for a period of two months we observed a striking increase in Prussian-blue staining, approximately five times that observed in either the control group or the mice immunized for one month (Fig. 5, *p *< 0.001). Following this initial increase in Prussian-blue staining, we observed a linear increase in staining associated with increasing duration of anti-Aβ antibody treatment (Fig 5). Five months of anti-Aβ antibody treatment demonstrated a six-fold increase in Prussian-blue staining when compared the control groups (Fig. 5).

## Discussion

Earlier studies with vaccines against the Aβ peptide demonstrated protection from the learning and memory deficits associated with amyloid accumulation in APP-transgenic mice [14,19]. Passive immunization protocols with anti-Aβ antibodies also produced cognitive benefits, in some cases even in the absence of significant reduction in amyloid burden [12,13]. Our recent work found that 3 months of anti-Aβ treatment of 18-month-old APP-transgenic mice improved spontaneous alternation performance on the Y-maze [14]. In the present work we confirmed that passive anti-amyloid immunotherapy can reverse spatial learning deficits in APP-transgenic mice and that this benefit of immunotherapy is retained, even in aged mice (26 and 28 months old at testing) with long-established amyloid pathology.

Additionally, we describe a more rapid means of testing spatial reference memory to reveal learning and memory deficits in APP-transgenic mice. This two-day version of the radial arm water maze included greater spacing of individual trials (mice spent time in their home cage after every trial), combined with less spacing of aggregate trials (fifteen trials per day rather than four or five) to facilitate learning of platform location in the nontransgenic mice, with a clear absence of learning in the age-matched transgenic mice.

A substantial reduction in total Congophilic amyloid deposits was observed in old APP-transgenic mice treated with anti-Aβ antibodies for 2 or more months. This measurement of total Congo-red staining included both parenchymal and vascular amyloid staining. When we analyzed the sections for only vascular amyloid (CAA) we found that this measure was significantly increased following 2, 3 and 5 months of anti-Aβ antibody treatment. The remaining parenchymal amyloid load was almost completely eliminated with this antibody approach. Clearly, because total amyloid load was significantly reduced not all amyloid was shifted into the vessels; but, it appears that at least some of the Congophilic material was redistributed to the vasculature. At the present time the mechanism for this redistribution is unclear. However, one possibility is that the microglia associated with the antibody-opsonized amyloid, either by phagocytosis or surface binding, and transported the material to the vasculature, possibly in an attempt to expel it. We and others have shown evidence for microglial involvement in the removal of amyloid using both intracranial anti-Aβ antibody injections [11,21] and systemically administered anti-Aβ antibody treatment [14], as well as *ex vivo *studies [10,22]. Here we also report our impression that microglia surrounding CAA vessels in immunized mice expressed more CD45 than control transgenic mice. This increased expression could be due to either increased expression in the same number of microglial cells or an increased number of microglial cells in these animals. It is feasible that this microglial activation was simply in reaction to the presence of increased amyloid in the blood vessels. However, it is equally likely that microglia activated by the opsonized material migrated to the vessels for disposal of the amyloid.

Cerebral amyloid angiopathy (CAA) is defined as the deposition of congophilic material in meningeal and cerebral arteries and arterioles (capillaries and veins can also show CAA but less frequently), and it occurs to some extent in nearly all Alzheimer's disease patients [23]. Severe CAA, affecting about 15% of cases, can be associated with both infarction and hemorrhagic injury [24,25]. It has also been shown that the severity of CAA can be directly linked to the severity of dementia in Alzheimer's disease patients [26].

In the current study we found a significantly increased number of microhemorrhages in the brain as detected by Prussian-blue staining, associated with the increase in CAA following passive immunization. Another transgenic mouse model of amyloid deposition, the APP23 mice, have been shown to deposit amyloid in both brain parenchyma and blood vessels and show a CAA associated increase in spontaneous cerebral hemorrhages [27]. Moreover, Pfeifer *et al*. [16] showed that these spontaneous hemorrhages were significantly increased following 5 months of passive immunization of 21-month-old APP23 mice using an anti-Aβ antibody with an N-terminal epitope, similar to those typically developed in active immunization with vaccines [4,28,29]. When young mice (6 months of age) were immunized following the same protocol, no hemorrhages were observed. More recently, DeMattos *et al*. [30] showed that passive immunization with an N-terminal antibody (3D6: directed against amino acids 1–5 of Aβ) of PDAPP transgenic mice also resulted in significantly increased microhemorrhage. They were unable to detect increased microhemorrhage with a mid-domain antibody (266: directed against amino acids 13–28 of Aβ). Notably, antibody 266 fails to bind Aβ deposited in CAA vessels or amyloid plaques [31]. Importantly, Ferrer *et al*. [7] noted the presence of CAA and microhemorrhage in the brain of one patient that participated in the Aβ-vaccine trial, even though the parenchymal amyloid appeared lower than expected. Also, Nicoll *et al*. [6] noted that CAA appeared unaffected in the brain of another patient that participated in the Aβ-vaccine trial.

It remains to be determined whether these observations regarding increased CAA and microhemorrhage in transgenic mice are relevant to trials of passive immunotherapy in humans. It should be noted that, in spite of extending the period of immunotherapy to 5 months, there was no discernable loss of the cognitive benefits of immunotherapy in the transgenic mice, all of whom showed increased microhemorrhage. While the observation that antibody 266 does not result in vascular leakage encourages testing of this idiotype, data from the Zurich cohort of the Aβ vaccine trial argue that brain-reactive antibodies may be important for cognitive benefits [32].

## Conclusions

Our opinion is that these results suggest that passive immunotherapy against Aβ should proceed with appropriate precautions taken to minimize the risk of hemorrhage (e.g., by excluding patients taking anticoagulants) and instituting measures to detect such hemorrhages if they do occur, irrespective of the antibody specificity or proclivity for microhemorrhage in aged APP-transgenic mice.

## List of abbreviations

Aβ : Amyloid-beta.

APP: Amyloid precursor protein

CAA: Cerebral amyloid angiopathy.

IgG1: Immunoglobulin G type 1.

## Competing interests

The authors declare that they have no competing interests.

## Authors' contributions

DMW treated the mice, performed the behavioral analysis, processed the tissue and performed pathological analyses, and drafted the manuscript. ARojiani evaluated slides and provided expert opinion regarding CAA and microhemorrhage. ARosenthal and SS developed, produced and purified the antibodies used in the studies. MJF performed DNA extraction and PCR for genotyping of the mice. MNG oversees the breeding colony generating mice for the studies, collected samples from the mice and assisted in editing the manuscript. DM conceived the design of the study, guided data interpretation and assisted in editing the manuscript.
